# “Be honest and gain trust”: a population health study to understand the factors associated with building trust in local government related to COVID-19 and vaccination in three historically disinvested neighborhoods in New York City

**DOI:** 10.3389/fpubh.2023.1285152

**Published:** 2023-10-25

**Authors:** Lauren J. Shiman, Fatoumata Diallo, Christina I. Nieves, Brandon Brooks, Rachel Dannefer, Sheena Dorvil, Maria Lejano, Jennifer Pierre

**Affiliations:** ^1^Bureau of Bronx Neighborhood Health, Center for Health Equity and Community Wellness, New York City Department of Health and Mental Hygiene, Bronx, NY, United States; ^2^Bureau of Harlem Neighborhood Health, Center for Health Equity and Community Wellness, New York City Department of Health and Mental Hygiene, New York, NY, United States; ^3^Bureau of Brooklyn Neighborhood Health, Center for Health Equity and Community Wellness, New York City Department of Health and Mental Hygiene, Brooklyn, NY, United States; ^4^Division of Epidemiology, New York City Department of Health and Mental Hygiene, Queens, NY, United States

**Keywords:** COVID-19, vaccines, trust, local government, population health

## Abstract

**Background:**

Distrust in government among people of color is a response to generations of systemic racism that have produced preventable health inequities. Higher levels of trust in government are associated with better adherence to government guidelines and policies during emergencies, but factors associated with trust and potential actions to increase trust in local government are not well understood.

**Methods:**

The COVID-19 Community Recovery study sampled participants from the New York City (NYC) Department of Health and Mental Hygiene’s NYC Health Panel, a probability-based survey panel who complete health surveys periodically. Participants who lived in one of three historically disinvested communities in NYC where the NYC Department of Health and Mental Hygiene has dedicated resources to reduce health inequities were included. The cross-sectional survey was fielded from September 30 to November 4, 2021 and could be self-administered online or conducted via CATI (Computer Assisted Telephone Interviewing) in English, Spanish, and Simplified Chinese (Mandarin and Cantonese by phone). Demographic data were summarized by descriptive statistics. Crude and adjusted logistic regression analyses were used to assess factors predictive of trust in local government as a source of information about COVID-19 vaccines. Open-ended responses about strengthening residents’ trust in local government were coded using an iteratively generated codebook.

**Results:**

In total, 46% of respondents indicated NYC local government was a trusted source of information about COVID-19 vaccines, relatively high compared to other sources. In bivariate analyses, race/ethnicity, age group, educational attainment, length of time living in NYC, and household income were significantly associated with identifying NYC government as a trusted source of information about COVID-19 vaccines. In multivariable logistic regression, no variables remained significant predictors of selecting local government as a trusted source of information. Key recommendations for local government agencies to build residents’ trust include communicating clearly and honestly, addressing socioeconomic challenges, and enhancing public COVID-19 protection measures.

**Conclusion:**

Study findings demonstrate that nearly half of residents in three historically divested NYC communities consider local government to be a trusted source of information about COVID-19 vaccines. Strategies to increase trust in local government can help reduce community transmission of COVID-19 and protect public health.

## 1. Introduction

Distrust of government entities among people of color is a response to generations of systemic racism that have produced preventable health inequities (1). Government-sanctioned policies, including redlining, although now federally banned, may still be practiced by institutions and have had subsequent and pervasive harms (2, 3). The effects of structural racism have negatively impacted housing quality, school funding, accumulation of intergenerational wealth, and other conditions that fuel a disproportionate burden of poor health outcomes and lower life expectancy in some urban neighborhoods with a higher proportion of people of color (4, 5).

To redress these injustices and to work in collaboration with community partners and residents to build healthier neighborhoods, the New York City Department of Health and Mental Hygiene (NYC Health Department) operates three place-based Bureaus of Neighborhood Health (BNH), which serve and are physically located in historically disinvested neighborhoods in North and Central Brooklyn, South Bronx, and East and Central Harlem (6). The NYC Health Department BNH are housed in spaces with co-located social service providers or clinical partners, and offer direct programming to residents as well as ongoing partnership and support to community partners. Residents of these neighborhoods are primarily Black and Latino (7). The COVID-19 pandemic has had disproportionate cumulative effects in these neighborhoods, including high rates of death due to COVID-19. From the start of the pandemic in February 2020 to the collection of data considered in this paper in October 2021, the age-adjusted COVID-19 mortality rates within the BNH catchment areas exceeded the citywide average (Brooklyn BNH: 387 per 100,000 people; Bronx BNH: 444; Harlem BNH: 325 compared to NYC average: 271) (8). Due to the legacy of structural racism and other injustices, residents of these three neighborhoods were already experiencing disproportionately high rates of chronic and infectious diseases prior to the onset of the COVID-19 pandemic (9). The disproportionate burden of COVID-19 cases, hospitalizations, and deaths drew renewed attention to the local inequities caused by this legacy.

COVID-19 prevention and mitigation efforts in these neighborhoods were a continuation and expansion of existing strategies to address broader health issues, including bi-directional communication with trusted messengers such as community based-organizations, faith-based leaders, school administrators, and other community leaders. These channels of communication helped to provide the NYC Health Department with important insight about residents’ fears and misconceptions, and simultaneously allowed accurate and timely health messages to be disseminated to residents, which are key elements to fostering trust between community and government (10, 11). This work was complemented by another essential tenet of the emergency response: direct communications from the NYC Health Department in the form of Public Service Announcements, public transit campaigns, regularly televised press conferences, webinars, in-person presentations at churches and other local gathering sites, street canvassing, and other outreach activities.

Simultaneously, misinformation and conspiracy theories related to SARS-CoV-2 and the COVID-19 vaccines grew, and gained traction on social media platforms (12). Anti-vaccination groups actively worked to develop distrust, capitalizing on the fear and worry of vaccine side effects (13). Believers of conspiracies tend to distrust government and scientific messaging and use conspiracies to create explanations for occurring problems. For marginalized communities, conspiracies can also stem from historical manifestations of racism in the form of institutionalized abuse towards that community (14). Perceived speed at which vaccines were developed and other specific concerns contributed to overall hesitancy to take the COVID-19 vaccines (15).

One study found that during the first year of the COVID-19 pandemic, Americans’ trust in government declined; decrease in trust was most pronounced among women, individuals who identified as Republicans, Black Americans, and individuals with lower educational attainment (16). Another study found that trust in government related to information about COVID-19 is associated with age, political party affiliation, race, and religious affiliation; this study found that Black Americans had the lowest levels of trust in government compared to other races (17).

Distrust in government can hinder public health efforts, particularly during large-scale emergencies such as the COVID-19 pandemic when government and healthcare institutions are rapidly issuing emerging guidance and instituting emergency measures (18). Guidance was also sometimes contradictory as the situation changed and new things were learned, such as changing guidelines around mask wearing early in the pandemic when the airborne nature of COVID-19 was not well understood. Trust in state and local government has been found to be associated with adhering to COVID-19 protective measures including mask-wearing and social distancing (17). Distrust in government may also contribute to poor mental health and burnout among public health professional; the national Public Health Workforce Interests and Needs Survey found that 28% of employees in 2021 had been challenged or undermined by non-experts (19). Therefore, building trust in government through transparent, timely, consistent, and meaningful efforts to improve local conditions and community health is a critical underpinning of a successful – and equitable – emergency response (11).

Valuable research has contributed to our understanding of why individuals may refuse the COVID-19 vaccination specifically, and how trusted messengers can increase uptake of the vaccine (20). However, less is understood about what factors contribute to trust in local government among residents of historically excluded communities. Therefore, it is important to understand how to build upon that trust to better serve these communities during future health emergencies and routine public health efforts.

This paper presents findings from the COVID-19 Community Recovery Survey conducted in three historically disinvested NYC communities where the local public health department has been working for several decades to build trust and credibility. Because these neighborhoods are similar with respect to demographic composition, historical disadvantage, and having a physical presence of and increased investment from the local health department, the neighborhoods are considered in aggregate as the BNH catchment area in all analyses. This paper explores demographic, social, and economic characteristics associated with reporting local government sources as trusted sources of information about COVID-19 vaccines, presents recommendations from the community to increase trust in local government, and considers the implications for these findings for urban health departments in the United States.

## 2. Methods

### 2.1. Participant recruitment

**Figure 1:**
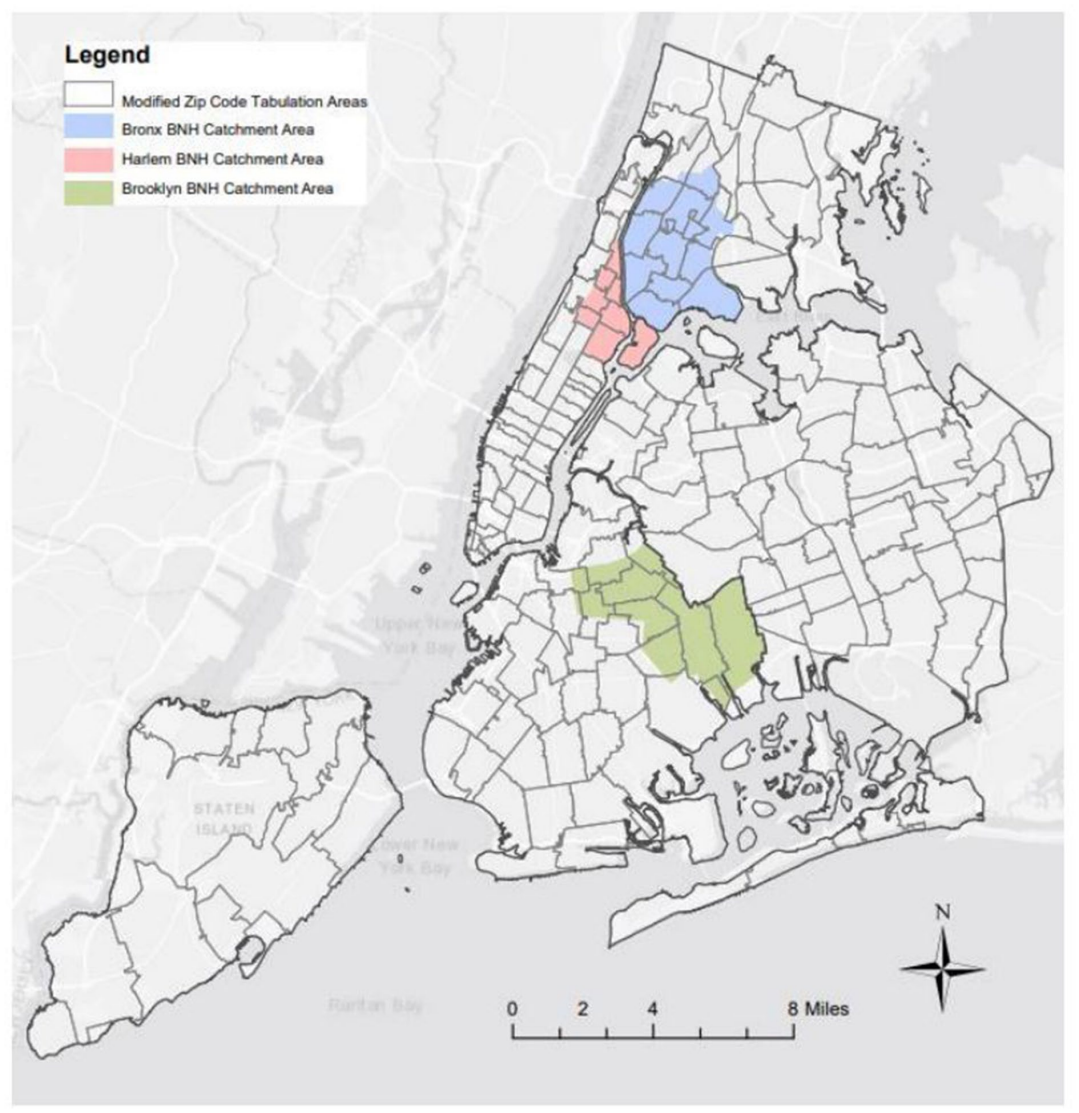
Geographic area surveyed in COVID-19 Community Recovery Survey.

COVID-19 Community Recovery study participants were recruited from the NYC Health Panel, a probability-based survey panel established in 2020 to supplement existing population-based health surveys (21). All panel members were 18 years or older and lived in NYC. At the time of the survey there were approximately 13,000 panel members. All 4,478 members who lived in one of 12 Community Districts or one of 25 ZIP code tabulation areas of the three BNH catchment areas were invited by mail, email, and/or text to participate. The geographic area included in the study is illustrated in Figure 1.

Each eligible NYC Health panelist received between three and six invitations to encourage participation. Method of invitation was based on the contact information that was provided during the NYC Health registration survey (email, mail and/or text). The survey was open from September 30, 2021, to November 4, 2021, and was electronically self-administered or conducted via CATI (Computer Assisted Telephone Interviewing) by a trained NYC Health Department interviewer. Participants provided informed consent verbally for interviewer-administered surveys and in written form for electronic surveys. Interviewers also phoned participants who did not respond to previous survey invitations to boost participation. All participants who completed the survey were offered a $10 gift card. Both self-administered and CATI surveys were offered in English, Spanish, and Simplified Chinese (Mandarin and Cantonese by phone).

### 2.2. Measures

The COVID-19 Community Recovery Survey questions spanned seven broad domains: impact of the COVID-19 pandemic on general healthcare, prescriptions, and mental health; attitudes towards COVID-19 vaccines and knowledge of NYC COVID-19 testing services; trusted sources of information for the vaccine; perceived community resilience and assets needed for recovery; trust in local government; social determinants of health; and familiarity with their local BNH office building. Participant demographics were collected during the initial NYC Health registration survey in either June 2020, September 2020 or May 2021; additional measures were collected at the time of the COVID-19 Community Recovery survey for variables that might have changed over time (e.g., zip code, gender). Trust in local government as a source of information about COVID-19 vaccines was measured with the following multiple select item: *“Where have you gotten information about the COVID-19 vaccines that you trust?”* Ten response options were provided, along with an open-ended *“other source”* option and the exclusive option *“There is no information you trust.”* Key findings from the trust in local government domain are presented in this paper.

### 2.3. Weighting and analysis

Detailed methods of the NYC Health panel construction (formerly known as Healthy NYC), as well as survey weighting and analytic methods of the COVID-19 Community Recovery Survey, have been described previously (21). Briefly, survey data were weighted to the residential adult population in the respective geographic area of interest to account for selection bias and nonresponse bias in analyses about trusted sources of information about COVID-19 vaccines. A survey respondent’s final weight is the product of several factors, including the initial probability of selection from the panel, nonresponse adjustments, pooling factors, and calibration. Demographic data were summarized by descriptive statistics; unweighted percentages and 95% Confidence Intervals (CIs) were reported (Table 1). Bivariate logistic regression models were used to measure the crude association between considering local government as a trusted source of COVID-19 vaccine information and participant demographics. All analytic models use survey weights to ensure the study sample reflects the weighted distribution of characteristics in the Bureau of Neighborhood Health catchment area neighborhoods. The following participant demographics were included: race/ethnicity, age group, individual educational attainment, birthplace, years lived in NYC, household language, household poverty status, public housing status, and vaccination status. Due to the low rate of missing data (<4%), a complete case analysis was conducted under the assumption that missing data was missing at random. Demographics that were significantly associated with local government as a source of trusted information in bivariate analyses were included in a multivariable model. A significance level of *p* < 0.05 was used to determine statistical significance. Model diagnostics were assessed to ensure no assumptions were violated. Open-ended responses about how local government can strengthen residents’ trust were coded using an iteratively generated codebook, initially developed based on the first 200 responses and revised to capture new themes as they emerged. Each response was coded separately by a primary and secondary qualitative analyst (LJS and FD) who then met to discuss any disagreements in coding and come to consensus. Each response was coded with all applicable codes; many responses are included in multiple themes. Quantitative analyses were conducted in SAS Enterprise Guide 7.15. Qualitative analyses were conducted in Microsoft Excel.

**Table 1:** Demographic characteristics of COVID-19 Community Recovery Survey participants; *N* = 1,358.

	*N*	%
BNH catchment area		
Brooklyn	401	29.5
Bronx	434	32.0
Harlem	523	38.5
Age group		
18–24	51	3.8
25–44	565	41.9
45–64	443	32.8
65+	291	21.6
Race/ethnicity		
Latino/Hispanic	508	38.5
Black, non-Latino	435	32.9
White, non-Latino	258	19.5
Asian non-Latino	73	5.5
Other/Multi-race, non-Latino	47	3.6
Gender		
Woman	968	71.5
Man	362	26.8
Transgender man, transgender woman, non-binary person, or other gender not mentioned	23	1.7
Individual education attainment		
Less than high school degree	169	12.5
Grade 12 or GED (high school graduate)	268	19.8
College 1 year to 3 years (some college, technical school, or associate’s degree)	335	24.7
College 4 years or more (college graduate)	299	22.1
Graduate degree or professional degree	284	21.0
Birthplace		
United States, excluding U.S. territories	853	63.3
Puerto Rico, Guam, U.S. Virgin Islands or other U.S. territory	74	5.5
Outside of the United States	420	31.2
Years lived in NYC		
Less than 5 years	115	8.5
5 to 10 years	124	9.2
More than 10 years	1,110	82.3
Household language		
English-speaking only household	741	54.9
Multi-lingual or non-English speaking household	609	45.1
Household poverty status*		
Annual income <200% Federal Poverty Level	661	52.4
Annual income ≥200% Federal Poverty Level	601	47.6
Lives in public housing (NYCHA^†^)		
Yes	254	19.4
No	1,057	80.6
Vaccination status		
Received at least one dose of a COVID-19 vaccine	1,277	91.2
Has not received a COVID-19 vaccine	119	8.8

^*^Poverty status was determined relative to 200% of Federal Poverty Level given the high cost of living in NYC. In 2021, 200% Federal Poverty Level was $53,000 for a household of four people. ^†^New York City Housing Authority.

This project was reviewed and deemed exempt research by the NYC Health Department’s Institutional Review Board.

## 3. Results

### 3.1. Study participants

Of the 4,478 invited NYC Health panelists, 1,358 unique participants (30.3%) completed the COVID-19 Community Recovery Survey online (*n* = 1,181) or through CATI (*n* = 177). Demographic characteristics of all survey participants are presented in Table 1. Among the 1,358 survey respondents, 29.5% lived in the Brooklyn BNH catchment area, 32.0% in the Bronx BNH catchment area, and 38.5% in Harlem. Most participants were Latino or Black (38.5% and 32.9%, respectively); ages 25–64 years (74.7%); had at least a high school degree (87.6%); and lived in households that were English-speaking only (54.9%), had income less than 200% of the Federal Poverty Level (52.4%), and were not in public housing (80.6%). Of note, 91.2% of the study sample reported having received at least one dose of the COVID-19 vaccine, compared to 82.7% of adult NYC residents who had received at least one dose as of October 1, 2021 (22).

### 3.2. Government as trusted source of COVID-19 vaccine information

As shown in Table 2, the most frequently reported source of trusted information about COVID-19 vaccines was *a doctor or other health professional* (54%), followed by *NY State and Federal (CDC) government* (47%) and *NYC government* (46%). Other common responses included *television news channel* (35%), *friends and family* (33%), *tv ads* (20%), *newspapers* (20%). Only 11% of respondents selected *radio,* 10% selected *social media* and 7% selected *community religious leader*. Six percent reported that there is no information they trust, and 7% listed another source.

**Table 2:** Frequency of reported source of trusted information about COVID-19 vaccines, age-adjusted and weighted to the residential adult population in the respective geographic area of interest.

Trusted sources of COVID-19 vaccines	Weighted #^†^	%	95% CI
Friends and family	327,500	33.2	(29.1–37.5)
Community religious leader (such as a pastor, priest, minister, rabbi, or imam)	65,800	6.6	(4.9–8.9)
A doctor or other health professional	537,700	54.8	(50.1–59.3)
NYC government (website, social media, or printed materials)	453,600	45.3	(40.8–49.9)
NY State and federal (CDC) government (website, social media, or printed materials)	466,400	46.8	(42.3–51.4)
Newspapers (online or in print)	194,200	19.1	(16.2–22.3)
Television news channel	349,500	35.3	(31.4–39.3)
Radio	107,500	10.5	(8.1–13.6)
TV ads	199,400	20.0	(16.7–23.9)
Social media	100,700	10.5	(7.9–13.7)
Facebook (WRITE IN)	24,700	23.9	(15.6–34.8)
Twitter (WRITE IN)	7,600	6.1*	(3.1–11.6)
Instagram (WRITE IN)	11,300	9.7*	(5.1–17.7)
Other social media	16,200	16.3	(8.9–27.7)
Other source	63,800	6.3	(4.5–8.6)
There is no information that I trust	62,800	5.7	(4.1–7.9)

^*^Estimate should be interpreted with caution. Estimate’s Relative Standard Error (a measure of estimate precision) is greater than 30%, or the 95% Confidence Interval’s half width is greater than 10, or the sample size is too small making the estimate potentially unreliable. ^†^Rounded to the nearest 100.

Bivariate logistic regression model results are reported in Table 3. Race/ethnicity and age group were significantly associated with selecting NYC local government as a trusted source of information about COVID-19 vaccines (*p*-value = 0.003 and 0.011, respectively). Black, non-Latino participants (OR = 0.43; 95% CI: 0.26, 0.70) and Latino/Hispanic participants (OR = 0.54; 95% CI: 0.34, 0.87) had lower odds of considering NYC local government as a trusted source compared to white participants. Living in NYC for more than ten years was associated with decreased odds of trusting local government (living in NYC >10y compared to less than 5y: OR = 0.70; 95% CI: 0.35, 1.38; *p*-value = 0.044). Those with higher levels of educational attainment had increased odds of trusting NYC local government compared to those with less than a high school degree (college graduate compared to less than high school degree: OR = 3.29; 95% CI: 1.93, 5.61; graduate or professional degree compared to less than high school degree: OR = 3.74; 95% CI: 2.13, 6.58; *p*-value <0.001). Living in a household with an annual income at or above 200% of the Federal Poverty Level was also predictive of trusting local government compared to living in a household with income below 200% (OR = 1.54; 95% CI: 1.06, 2.24; *p*-value = 0.024).

**Table 3:** Crude and adjusted odds ratios of reporting NYC Local Government as trusted source of information about COVID-19 vaccines.

	Crude OR (95%CI)	*p*-value	Adjusted^§^ OR (95% CI)	*p*-value
Age group (n_c_ = 1,265; n_a_ = 1,137)^†^				
18–24 years	Ref	0.011*	Ref	0.277
25–44 years	1.64 (0.70, 3.85)		2.00 (0.77, 5.17)	
45–64 years	1.09 (0.46, 2.54)		1.75 (0.69, 4.48)	
65+ years	0.75 (0.31, 1.81)		1.30 (0.49, 3.43)	
Race/ethnicity (n_c_ = 1,237; n_a_ = 1,137)				
White, non-Latino	Ref	0.003*	Ref	0.308
Black, non-Latino	0.43 (0.26, 0.70)		0.71 (0.38, 1.34)	
Latino/Hispanic	0.54 (0.34, 0.87)		1.07 (0.58, 1.95)	
Asian, non-Latino	1.18 (0.54, 2.56)		1.54 (0.62, 3.84)	
Other/Multi-race, non-Latino	0.79 (0.27, 2.34)		1.03 (0.38, 2.77)	
Gender (n_c_ = 1,267)				
Man	Ref		N/A	
Woman	0.94 (0.65, 1.37)	0.541	N/A	
Transgender man, transgender woman, non-binary person, or other gender not mentioned	1.98 (0.50, 7.89)		N/A	N/A
Individual educational attainment (n_c_ = 1,269)				
Less than high school degree	Ref	< 0.001*	Ref	0.064
High school graduate	1.67 (0.97, 2.93)		1.81 (0.97, 3.36)	
Some college, technical school, or associate’s degree	1.73 (1.04, 2.89)		1.68 (0.92, 3.07)	
College graduate	3.29 (1.93, 5.61)		2.45 (1.24, 4.82)	
Graduate degree or professional degree	3.74 (2.13, 6.58)		2.84 (1.35, 5.96)	
Birthplace (n_c_ = 1,262)				
United States, excluding U.S. territories	Ref	0.971	N/A	N/A
Puerto Rico, Guam, U.S. Virgin Islands or other U.S. territory	0.94 (0.51, 1.74)		N/A	
Outside of the United States	0.96 (0.66, 1.41)		N/A	
Years lived in NYC (n_c_ = 1,264; n_a_ = 1,137)				
Less than five years	Ref	0.044*	Ref	0.565
Five to ten years	1.43 (0.60, 3.40)		1.63 (0.66, 4.04)	
More than ten years	0.70 (0.35, 1.38)		1.33 (0.64, 2.78)	
Household language (n_c_ = 1,264)				
English-speaking household only	Ref	0.595	N/A	N/A
Multi-lingual or non-English speaking household	1.10 (0.77, 1.57)		N/A	
Household poverty status (n_c_ = 1,264; n_a_ = 1,137)				
Annual income <200% Federal Poverty Level	Ref	0.024*	Ref	0.552
Annual income ≥200% Federal Poverty Level	1.54 (1.06, 2.24)		1.14 (0.74, 1.76)	
Lives in public housing (NYCHA^‡^) (n_c_ = 1,229)				
Yes	Ref	0.786	N/A	N/A
No	0.94 (0.62, 1.43)		N/A	
Vaccination status (n_c_ = 1,262)				
Received at least one dose of a COVID-19 vaccine	Ref	0.829	N/A	N/A
Has not received a COVID-19 vaccine	0.92 (0.44, 1.93)		N/A	

*Significant at *p*-value < 0.05 level. ^†^nc = sample size for crude mode; na = sample size for adjusted model; missingness in the data was < 4%. ^‡^New York City Housing Authority. ^§^Model adjusted for age group, race/ethnicity, individual educational attainment, years living in NYC, household poverty status, living in public housing, and vaccination status.

In the multivariable logistic regression model, no independent variables remained significantly associated with selecting NYC local government as a trusted source of information about COVID-19 vaccines (Table 3), potentially in part due to the interrelated nature of some demographic variables (i.e., living in public housing and household poverty below 200% of Federal Poverty Level).

### 3.3. Ways to strengthen trust in local government

Survey participants responded to the open-ended question, *“During this stage of the pandemic, what should the local NYC government do to strengthen your trust in it?”* Out of 1,358 total survey participants, *n* = 144 responded with “NA”; *n* = 80 responses were not codable; *n* = 51 responded that they did not know; *n* = 237 did not respond to this question. The remaining 846 (62.3%) provided a codable response to this question, including that they already trusted the government (*n* = 101), that the government could not be trusted regardless of any attempts to strengthen trust (*n* = 24), and with suggestions to strengthen trust in government. Key themes and subthemes about how to strengthen trust emerged from the codable responses; these themes are presented in Table 4. The most common themes are presented in more detail below.

**Table 4:** Key themes from residents’ suggestions to strengthen trust in local government.

Theme	Frequency (*n*)	Subthemes	Illustrative quote
Communicate clearly and honestly	316	Share information	“Be transparent with statistics and new information.”
		Be truthful	“Be honest and gain trust.”
		Change communication strategies	“I think the government assume[s] everyone has a TV or some form of media to see the constant barrage of information. I think there should be info given out at transit hubs or bus and train stations or any other place people congregate.”
		Be consistent in messaging and actions	“Every outlet should have been on the same page. The governor was saying one thing and the mayor would say something completely different.”
Address socioeconomic challenges	144	Address housing	“More rent support.”
		Address public safety	“Enforce public safety in MTA subways.”
		Provide financial support	“Give another stimulus check to help pay bills and get more food.”
		Address unemployment	“Employment or getting people help that are still unemployed.”
		Provide food resources	“Give people food.”
Enhance public COVID-19 protection measures	91	Increase/continue protective policies (e.g., mask mandates, vaccine requirements)	“…They should have kept the mandatory mask[s] cause it’s spreading without people wearing…mask[s].”
		Increase enforcement of existing protective policies (e.g., masks on public transit)	“Enforce mask wearing on public transportation.”
Increase vaccination rates	80		“Not to let their guard down. Keep pushing for higher vax rates.”
Increase/continue local outreach	74	Community engagement	“Keep reaching out to the public and community leaders.”
		Be visible	“Be more present.”
Protect vulnerable populations (e.g., older adults, low-income families, people experiencing homelessness)	53		“What they can do is check on the older population. My neighbor, I have to buy her groceries because she is old and does not want to go outside with all the COVID. Check on who is old, who needs help, bring them groceries like in early COVID…”
Take responsibility	44	Model behaviors	“Wear masks where the public is required to wear masks and take City COVID regulation enforcement more seriously.”
		Accountability for officials	“At the end of the pandemic, whenever that may be, I think the local government should acknowledge the mistakes they made and map out a plan for future pandemics.”
Enact other policy change (e.g., bail reform, increased paid sick time, sanitation, immigration policy)	38		“Stop evicting and deporting undocumented people.”
Follow science	30		“Focus less on economic factors and more on science.”
Provide general support	29		“Have more help for the community.”
Decrease public COVID-19 protection measures	27		“Accept… freedom of choice and stop mandating vaccinations.”
Expand testing services	16		“Expand rapid testing at corner stores, bodegas, and churches.”
Change or keep specific school policies	16		“Make sure all teachers and staff are vaccinated (no excuses) and tested weekly.”
Address mental health issues	12		“Provide more funding for mental health.”
Provide PPE	5		“Get masks to every household at least once a week for free to everyone in the house.”
Support local businesses	5		“Continue making vaccination a mandate and helping stores and restaurants reinforce it.”
Improve COVID-19 vaccine efficacy	3		“Keep looking for a safe vaccine that would stop you from getting COVID even after the vaccine.”
Other response	n/a		n/a

### 3.4. Communicate clearly and honestly (*n* = 316)

Several subthemes emerged related to government communication with the public. Participants made general comments about the importance of consistent and frequent communication from local government, including appreciation for what was perceived as a lot of information shared throughout the pandemic and a desire for more information. Some responses indicated specific information to be shared, for example, *“Continue to share the number of cases and deaths daily.”* Others more generally described the importance of providing accurate information as the situation evolves, illustrated by the responses *“continue with updated scientific information as our knowledge develops about COVID”* and *“more explicit information about the science they are using to drive decisions”*.

Another subtheme emerged about the need for transparency and truthfulness. Some participants implied that government has been *“holding back information,”* and many indicated that being forthcoming with all information was necessary to build trust.

Participants also suggested changes to current communication strategies, such as waiting to release new information until it is confirmed, mailing out information, and more intentionally countering widespread misinformation.

Finally, participants encouraged better consistency across government messaging. Responses indicated that messaging *“about vaccines and boosters has vacillated,”* and that messaging has been inconsistent and confusing. Similarly, participants pointed out specific instances where actions felt contradictory to public messaging and potentially undermined the message. For example, one respondent said, *“Allowing people to go out and do things [that require vaccination] with a single dose of the vaccine although you are not fully vaccinated until you are two weeks after your second dose sends mixed messages”*.

### 3.5. Address socioeconomic challenges (*n* = 144)

Another theme was to address social and economic challenges in the neighborhood to strengthen trust in local government. Participants identified specific supports they expected of a trustworthy and well-functioning local government, especially related to housing, food, unemployment, public safety, and financial support. Participants emphasized the social and economic hardships exacerbated by the pandemic and expressed expectations that government should address the high costs of housing through rent relief or lowered property taxes, disrepair of rental units including public housing units, and predatory landlords; participants also identified the need for government to provide free groceries or other food resources and to provide direct financial support to individuals and families. Considerations for the most vulnerable were elevated: participants felt a trustworthy government would prevent evictions and providing housing to people experiencing homelessness. One respondent highlighted long-term benefits of more intensive government support to address socioeconomic issues: *“The local NYC government should be focused on providing affordable housing, basic income, food stamps, employment, childcare, healthcare,* etc. *to all people in NYC so that when the next pandemic hits, the general standard of living is higher”*.

### 3.6. Enhance public COVID-19 protection measures (*n* = 91)

Ninety-one participants wrote-in responses related to maintaining or increasing public measures of protection. Of these, approximately 30 participants explicitly expressed support for vaccine mandates at places of employment and at restaurants and other public spaces, and approximately 20 explicitly expressed support for mask requirements in public spaces. One person said that to build trust the government should *“stop rushing to get everything back to normal,”* while another suggested that a trustworthy local government should *“not give in to all the whining and complaining about vaccines, mask wearing and social distancing”*.

Other responses related to this theme focused on enforcing existing protective measures such as checking vaccination cards in businesses that required vaccines and enforcing masking requirements on buses and trains.

### 3.7. Other major themes

Other common themes include increase vaccination rates, increase/continue local outreach, protect vulnerable populations, and take responsibility. Respondents described that the local government should “*keep pushing for higher vax rates*,” engage with and be visible in the community, and “*continue to take care of the people who have been the worst affected*.” The theme of take responsibility reflects two subthemes: model behaviors and accountability for officials. Model behaviors referred to government but especially to local law enforcement. Respondents advised that government and law enforcement should “*wear masks where the public is required to wear masks and take city COVID regulation enforcement more seriously*.” Responses that mentioned accountability for officials include holding elected officials accountable by voting them out in future elections if important promises are broken and that “*the local government should acknowledge the mistakes they made and map out a plan for future pandemics*”.

## 4. Discussion

Findings from a cross-sectional study in three historically disinvested neighborhoods in NYC demonstrate that 46% of adult residents in these communities consider local government to be a trusted source of information about COVID-19 vaccines. At a national level, preexisting data about trust in government is complex and often conflicting. In a large survey of Facebook users across 48 states, health professionals and scientists were listed among the most trusted sources of information about COVID-19 vaccines (23). However, national polls indicate that overall trust in government has remained relatively low over the past two decades: in April 2021, only 21% of Americans trust the government to do what is right “just about always” or “most of the time” (24). Moreover, misinformation about COVID-19 vaccines has permeated public perception, implying that social media and word of mouth are also believed sources of information. National data from the Kaiser Family Foundation COVID-19 Vaccine Monitor indicate that 80% of Americans believe to be true or are uncertain about at least one incorrect sentiment related to COVID-19 vaccines (25). Building trust between residents and their local government is a highly complex issue that the NYC Health Department, like many health agencies, continues to work towards and grapple with. These findings provide a baseline assessment of trust among residents in three historically disinvested neighborhoods specifically with respect to COVID-19 vaccine information which can be used as a point of comparison at future timepoints. They also provide an opportunity for NYC local government to learn from perceptions of the pandemic response, and strengthen communication and other strategies to build credibility and public trust, in preparation for future emergency response.

While significant resources have rightfully gone to supporting religious leaders and community-based organizations to promote accurate COVID-19 messaging, these findings imply the need for continued resources and support for direct government outreach, community engagement, and communication campaigns as information regarding public safety as the pandemic continues to evolve. Relationship development requires time and consistency. Through consistent physical presence of the NYC Health Department in these neighborhoods by the work of the Bureaus of Neighborhood Health, relationships between residents and local government, as well as community partners and local government, have been intentionally cultivated and likely contributed to the perception of local government as a trusted messenger on this topic. These findings provide support for the need for continued and consistent government investment and engagement in historically disinvested neighborhoods.

The bivariate results identify populations that are less likely to trust government about COVID-19 vaccines, including people with less than a college degree, Black and Latino residents, those living in NYC for more than ten years, residents living in low-income households, and those living in public housing. Respondents are from neighborhoods that have been subjected to generations of systemic disinvestment; lower educational attainment and poverty persist due to government policies that dictated mortgage lending practices and school funding (26–28). Results could help inform priority populations for consistent and meaningful outreach. The multivariable model results demonstrate a marginally non-significant association between educational attainment and trust in local government; given the weighted study population skewed towards lower educational attainment, further research is warranted to better understand the relationship between education and trust in government. Further, some demographic subgroups had small sample size (e.g., people identifying their race as non-Latino Asian, transgender and gender non-conforming people). These categories were intentionally not collapsed into other subgroups to avoid further erasure of already systemically excluded communities, but small sample size yielded wide confidence intervals. Intentional oversampling of underrepresented populations in future survey panels can support better understanding of the experiences of these groups.

Issues of vaccine hesitancy, vaccine confidence, and vaccine acceptance, are complex and nuanced. Prior to the approval of any COVID-19 vaccine, social media surveillance revealed that social media users living in New York or London were more likely than those in Mumbai, Beijing, or Sao Paolo to post about a lack of confidence in vaccine safety and to distrust government promotion of the COVID-19 vaccines (29). In practice, public policies that instill fear of government also played a role in acceptance; for example, fear of Public Charge among undocumented people was a barrier to accepting the vaccine even among those confident in the vaccine itself (30). Vaccine hesitancy is a dominant narrative portrayed specifically about the perceptions of communities of color (31). However, a recent study used thematic analysis to understand themes across stories of NYC residents in low-income neighborhoods who were uncertain about the COVID-19 vaccines but ultimately decided to accept the vaccine (32). Among key reasons for vaccine acceptance were a strong sense of social solidarity and the desire to have a positive impact in their communities. Better understanding the motivators for receiving COVID-19 vaccines can help to shape public communications that build, rather than undermine, trust. Further, public communications, supported by enforced policies, that emphasize community spirit rather than individualism may be most effective in improving community health, particularly as it concerns a highly transmissible virus that thrives on social interactions to spread.

Overwhelmingly, qualitative data highlighted the need for clear, transparent, and consistent communication from all government bodies to build trust in local government entities. Some of the strategies recommended by residents are already in place but responses identify a lack of visibility. Better coordination between government agencies and increased consistency between local, state, and federal messaging may help to build needed trust. Eliminating contradictory messaging was elevated as a key theme to build trust, corroborating findings from Van Scoy et al. (33). The Centers for Disease Control and Prevention issues guidance for effective emergency communication, but many key pillars of this model were disregarded during the COVID-19 pandemic (34). For example, the guidelines recommend allowing subject matter experts to deliver public communications rather than elected officials. However, during the early phases of the COVID-19 outbreak in NYC many health messages were delivered directly by the Governor or Mayor. Public perception that health decisions were made by officials without medical or public health credentials might contribute to distrust. The guidelines also reiterate the importance of consistent messaging and framing around the nature of constantly evolving information, but this framing was missing from many public communications about the state of the COVID-19 pandemic and specifically the implications of vaccination (e.g., the shift from the narrative that vaccines will prevent against transmission to “breakthrough infections” to vaccines as protection against severe disease rather than infection). Critically evaluating COVID-19 related public health communications, revisiting tested methods of emergency communication, and recommitting to best practices is essential in preparation for future health emergencies.

Respondents also highlighted the need for government to address social and economic challenges to build trust. Based on responses, some participants appear to conflate the powers of local and federal government, for example by requesting additional stimulus checks from local government. However, the responses clearly indicate that a trustworthy government will ensure that the basic needs (e.g., food, shelter) of its constituents are met; systemic disinvestment in these specific neighborhoods likely exacerbates the need for government support related to socioeconomic concerns. Realistic mechanisms for government to provide basic needs to impoverished communities in NYC requires deliberate consideration. Policies that decrease food insecurity (e.g., government-funded food as medicine programs), and increase economic stability (e.g., universal basic income, increased living wage) could bolster trust by allowing government to better meet basic needs of its constituents.

A key theme was support for prevention measures, such as vaccine and mask mandates, including that increasing such measures would increase trust in local government. Despite narratives in the media and perceptions of politicians, prior research corroborates these findings. A study conducted by the Pew Research Center in August 2021 found that 62% of participants reported that the health benefits of COVID-19 restrictions on public activity have been worth the costs (35). Similarly, qualitative data from this study elevated the expectation that government should protect vulnerable populations. Disproportionate media attention has been paid to school closures and restrictions on college campuses without nuanced discussion of the role infections in children played in household transmission to more vulnerable family members. Increased government attention to protecting older adult populations and those with chronic comorbidities, as well as increased media coverage of efforts that were made to protect the most vulnerable populations, may serve to build public confidence in the response. A desire for government to protect the most vulnerable is also at odds with the current communications to assess personal risk rather than having public policies in place to protect vulnerable members of a community. Policies that protect the health of vulnerable populations in public spaces, including updating the ventilation systems of public buildings to improve indoor air quality, convenient provision of no-cost masks to the public, and requiring masking in healthcare facilities or on public transit, may strengthen trust in government by demonstrating government-issued protections for at-risk community members.

The findings from this study present a snapshot of residents’ perspectives at a particular point in time during the COVID-19 pandemic. The context of the pandemic conditions at the time of data collection likely influenced perspectives of residents. The survey was conducted nearly twenty months after the first cases of COVID-19 were diagnosed in NYC, and during a period of lower community transmission between the peak of the Delta wave in Summer 2022 and the Omicron wave in Winter 2021. At the time of data collection, COVID-19 vaccines had been readily available in NYC for all individuals age five and older for several months, and as of October 1, 2021, 82.7% of adult NYC residents had received at least one dose of a COVID-19 vaccine (22). Notably, among our survey sample, 90.8% of respondents reported having received at least one dose of a COVID-19 vaccine.

Public policy also contributes to perspectives at a given point in time. New York State policy at the time of the survey required masks in public indoor spaces and the Key to NYC policy required NYC indoor venues including restaurants, fitness facilities, and entertainment spaces to check for proof of COVID-19 vaccination prior to entry (36). These policies were suspended on February 10 and March 7, 2022, respectively (37, 38). In May 2022, NYC reached a “high alert” level in the NYC Government’s own COVID alert system that intended to trigger renewed indoor masking requirements, but NYC Mayor Adams did not reinstate such requirements (39). When taken in the context of these study findings related to consistency in messaging and actions and increasing COVID-19 public prevention measures, it is possible that actions such as these could serve to lessen trust in local government.

### 4.1. Limitations

This study demonstrates associations between some demographic characteristics and trust in local government as a source of information about the vaccine, but as data are drawn from a cross-sectional study the ability to draw causal inferences is limited. Although the data were collected from a probability-based study panel, there are some limitations in generalizability particularly relevant to the findings presented in this paper. Most notably, neighborhood residents who opted to participate in a NYC Health Department survey may differ from other residents in important ways; as noted previously, the study sample was more likely to have received a dose of the COVID-19 vaccine than NYC residents overall at the time of data collection. As the panel is, by definition, residents willing to engage with the NYC Health Department, they may be more likely than residents who did not consent to join the panel to trust local government. Further, the neighborhoods included in this study differ from the overall population of NYC in several important ways, including demographic characteristics and historical context. As such, findings may not be generalizable to the broader NYC population. Additionally, because a complete case analysis was conducted, multivariable modeling may be subject to bias if the assumption that data were missing at random does not hold. Given the low level of missingness (<4%), the magnitude of potential bias is expected to be small. Finally, the perspectives of neighborhood residents who speak and read a language other than English, Spanish, or Chinese are not represented. Despite these limitations, the study contributes valuable information about the perspectives and recommendations of residents living in low-income communities about how to build trust in local government.

### 4.2. Conclusion

Study findings showed that there was a reasonably large proportion of residents in the three historically disinvested neighborhoods in NYC that viewed local government as a trusted source of information about COVID-19 vaccines, and that the long-standing relationships with the NYC health department is a factor that can be further leveraged to increase trust and coordination of vaccinations in these often-excluded communities. Resident feedback and suggestions, including those displayed in Table 4, serve as a potential roadmap for strategies that can be implemented to gain or increase public trust. Strategies to increase trust in local government include clear, transparent communication and providing government support to address social and economic challenges. Study participants supported government-enacted protective measures to reduce community transmission of COVID-19 and expected government to take such measures to protect themselves, vulnerable populations, and the City at large.

## Data availability statement

The datasets presented in this article are not readily available due to privacy restrictions. The data that support the findings of this study are available from authors upon reasonable request and with permission of NYC Department of Health and Mental Hygiene. Requests to access the datasets should be directed to LS at lshiman@health.nyc.gov.

## Ethics statement

All protocols were carried out in accordance with relevant NYC Department of Health and Mental Hygiene guidelines with respect to informed consent, data storage, and other considerations. This project was reviewed and deemed exempt research by the NYC Health Department’s Institutional Review Board (Protocol #21-053). Informed consent was received from all study participants at the time of the survey.

## Author contributions

LS: Conceptualization, Data curation, Formal analysis, Investigation, Methodology, Writing – original draft, Writing – review & editing. FD: Conceptualization, Data curation, Formal analysis, Investigation, Methodology, Writing – review & editing. CN: Conceptualization, Data curation, Formal analysis, Investigation, Methodology, Writing – review & editing. BB: Conceptualization, Investigation, Writing – review & editing. RD: Conceptualization, Data curation, Formal analysis, Investigation, Methodology, Writing – review & editing. SD: Conceptualization, Data curation, Formal analysis, Writing – review & editing. ML: Conceptualization, Funding acquisition, Investigation, Writing – review & editing. JP: Conceptualization, Investigation, Methodology, Writing – review & editing.

## Abbreviations

NYC health department: NYC department of health and mental hygiene
BNH: bureaus of neighborhood health
CATI: computer assisted telephone interviewing
CI: confidence interval
